# Investigation of Composition, Structure, Electrical Properties, and Ageing Resistance of Conductive Flocked Fabric for Automotive Applications

**DOI:** 10.3390/polym17162212

**Published:** 2025-08-13

**Authors:** Matilde Arese, Elio Sarotto, Antonino Domenico Veca, Vito Guido Lambertini, Daniele Nardi, Martina Sandigliano, Federico Cesano, Valentina Brunella

**Affiliations:** 1 Department of Chemistry, University of Turin, 10125 Turin, Italy; matilde.arese@unito.it (M.A.); elio.sarotto@unito.it (E.S.); federico.cesano@unito.it (F.C.); 2 Fiat Research Center SCPA (CRF), Stellantis, 10135 Turin, Italy; antonino.veca@crf.it (A.D.V.); vitoguido.lambertini@crf.it (V.G.L.); 3 Apollo Srl, 50013 Campi Bisenzio, Italy; daniele.nardi@sageai.com; 4 Technova Srl, 07026 Olbia, Italy; martina.sandigliano@technovaitalia.it

**Keywords:** e-textiles, flocked yarns, durability

## Abstract

The growing development of conductive functionalised textiles has attracted the interest of the automotive industry, which is seeking innovative solutions for seamless and futuristic interior design aimed at improving both vehicle aesthetics and user experience. In line with this trend, the present work investigates the electrical performances of two conductive flocked yarns, one incorporating silver-coated fibres and the other carbon black-based fibres, for potential application in smart automotive interiors. The stability of their electrical properties was also evaluated under thermal ageing and mechanical stress conditions. Thermogravimetric analysis (TGA), differential scanning calorimetry (DSC), and field emission scanning electron microscopy (FE-SEM) investigations provided information about the composition and structural properties of the yarns. Silver-based yarns demonstrated superior conductivity and thermal stability. In contrast, carbon-black yarns exhibited lower electrical performance and increased sensitivity to ageing due to filler agglomeration. A multitouch capacitive sensor prototype was also developed using the silver-based fabric and successfully integrated into a microcontroller platform. The results demonstrate the suitability of conductive flocked textiles for durable, low-voltage human–machine interfaces requiring robust, flexible, and responsive textile-based control surfaces, such as automotive applications, consumer electronics, and wearable technology.

## 1. Introduction

Over the last decades, electronic textiles (e-textiles) have attracted significant attention due to their ability to combine two key properties: flexibility and electrical conductivity. Their application spectrum is broad, first of all in healthcare [1]: Ali et al. developed a passive chest-strap sensor capable of real-time respiratory tracking and Bluetooth transmission of data to a smartphone, enabling early detection of critical conditions [2]. Other notable applications include yarn-based smart gloves with sign-to-speech translation ability [3], textile-based heart valve prostheses [4], chemical sensors for real-time monitoring of humidity [5], biofluid composition, and respiratory gases. In the sports sector, e-textiles assess physiological signals and biomechanics during exercise [6], such as garments equipped with an IMU sensor and EMG electrodes able to analyse neuromuscular responses to training [7]. Another example in the field is a smart bra that adapts its stiffness and strap tension based on breast movement to enhance comfort and reduce breast pain [8]. The military and energy-storage sectors also benefit [9,10]: screen-printed inks on military textiles have been used to fabricate wearable supercapacitors [11]. Expanding the context, conductive textiles, especially silver based fabric, also exhibit notable antibacterial properties. Silver nanoparticles (Ag NPs), when deposited onto cotton fibres, have demonstrated strong antibacterial activity against gram-negative bacteria such as *Escherichia coli* [12]. Moreover, Ag NPs can be utilised to develop bifunctional materials that are both conductive and antibacterial, making them suitable for use as electrodes in wearable electronic devices [13]. Conductive yarns can be incorporated into fabrics using traditional textile techniques such as weaving, knitting, and sewing, as well as through novel methods like coating, screen printing, and magnetron sputtering [14], depending on their final applications. Conductive fibres are typically composite materials, where the conductive elements can be either on the surface of insulating fibre (surface conductive fibres) or embedded inside the fibre (core conductive fibres) [15].

In the last few years, Original Equipment Manufacturers (OEMs) designers started focusing their attention on the integration of lightweight, multifunctional smart textiles in car interiors. These materials offer the potential to reduce vehicle weight by replacing conventional electronic systems, while also enabling advanced control of multimedia, windows, and climate systems through textile-based interfaces [16]. E-textiles represent a promising solution for reducing interfaces in the central console, embedding them in the interior to create a more dynamic and adaptable car interior, which could stimulate more pleasurable in-car interactions [17].

A large number of laboratory-scale conductive textiles for smart solutions can be found in the literature; however, their commercial adoption remains limited due to challenges related to reliability complications, electrical connectivity, long-term electrical performance, and problems related to interconnection of e-textile structures [18]. For applications under complex service conditions and environments like an automotive interior, soft sensors must offer both high sensitivity and a wide linear response range. Designing hierarchical structured layers and engineered microstructures in soft sensors is an effective approach to meet these demands [19]. A promising solution in this field is constituted by flocked fabrics. These are textiles are composed of substrate, typically a polyester/cotton blend, adhesive (e.g., water-based acrylic, polyurethane, or polyvinylchloride), and vertically oriented flock fibres, such as polyamide or viscose, aligned through the application of an electrostatic field [20,21]. The incorporation of fine nylon microfibres in the contact layer enhances tactile comfort, while also providing high durability and mechanical resistance [22,23]. Flocked fabrics are applied in different sectors, like sport and military, as energy-absorbing material [24], biomimetic pressure sensors [19,25], and wearable energy storage applications [26].

Indeed, the flock architecture itself provides insulation between adjacent conductive fibres and ensures protection of the conductive fibre from external stresses, making it an excellent solution for pressure-sensing applications in automotive interiors.

In this work, we evaluated the electrical performances of two flocked textiles, each realised with different types of conductive fibres: one covered with a homogeneous silver (Ag) coating [27] and the other with carbon black (CB) coating. Silver (Ag) is at the top of the list in electronic circuit applications because of its good balance between electrical conductivity, ease of integration into textiles, [28] and cost, compared to other metal fibres, such as Au. However, its high cost remains a significant limitation, leading to a growing interest in cheaper alternatives such as copper, aluminium, graphene, and, particularly, carbon black [29,30,31]. CB price is around 1EUR/kg and, in addition, it exhibits excellent electrical conductivity, it is not toxic, and it is easily dispersed in solvents [32]. In addition, in the literature, there were already developed flocked fabric modified using carbon black to achieve electrical conductivity properties [19].

We further evaluated the durability of these conductive textiles by subjecting them to various mechanical and thermal ageing tests to assess the resistance of the conductive fibres to mechanical damage and electrical conductivity loss. To the best of our knowledge, this study represents one of the first investigations of conductive flocked fabric for automotive application, including an evaluation of ageing resistance.

Thermal properties, morphology, and DC electrical conductivity were investigated to elucidate the different properties of each sample. Furthermore, samples were tested as multitouch sensors connected with conventional low-power electronics and controlled by inexpensive and commercially available microcontrollers such as Arduino MEGA 2560. A multitouch media and music control interface was produced as a working prototype.

### E-Textiles in Automotive Interiors: Enhancing Functionality and User Experience

The automotive industry is continuously in search of innovative and distinctive solutions capable of capturing the attention of the consumer and stand out from the other OEMs. To reach this objective, smart textiles were found to be among the most promising frontiers for future vehicles. These advanced materials not only enrich the functionality of automotive interiors but also align with eco-innovation principles by reducing the need for traditional mechanical components and contributing to weight reduction. A clear example is the research published by Grancaric et al [33]., in which they developed conductive inks that can be printed on flat plastic surfaces and molded into three-dimensional components, replacing bulky wired systems and reducing both weight and dimensions.

The integration of smart textile materials in seats, armrests, and door panels allows for the replacement of conventional buttons and control interfaces. This results in more streamlined, aesthetically pleasing, and revised car interiors matching both interaction design and interior design into a seamless union [17].

From a functional perspective, textile-based sensors and conductive fibres are being employed to monitor the thermal comfort [34], air quality, driver fatigue, or passenger stress levels [35]. Moreover, the novelties in car interiors are inclined to propose the e-textile input interfaces as tactile screenless means of controlling multimedia, windows, and A/C inside the car. Extensive progress has also been conducted on light-emitting textiles [36], fabric audio speakers [37], and shape-changing fabrics [38]. This enables the vehicle to become not just a means of transportation, but an interactive and adaptive living space, especially relevant in the context of autonomous driving and shared mobility [16].

## 2. Materials and Methods

### 2.1. Materials

**Figure 1:**
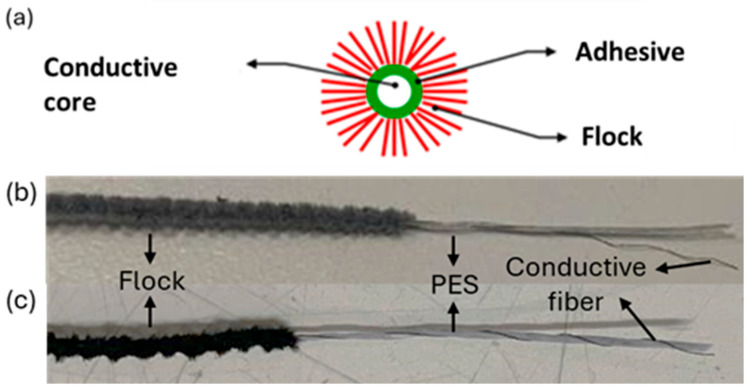
(**a**) Schematic illustration of the conductive flocked yarn structure, composed of a conductive core, adhesive layer, and flock fibres; (**b**) F-Ag internal structure: it is possible to distinguish the flock in a grey colour; the core is constituted of PES (white yarn) and the conductive fibre in grey; (**c**) F-CB internal structure: it is possible to distinguish the flock in black colour, and the core is constituted of PES (white yarn) and the conductive fibre in black.

Flocked yarns were produced by Technova S.r.l. (Olbia, Italy). The first sample chosen for the study was a flocked yarn made by polyamide (PA) 6.6, with a linear density of 1.9 dTex (1.9 g per 10,000 m of fibre) and a diameter of 0.65 mm. The core was made with 730 dTex PES (polyester) (97.5%) yarn and PA 6 (2.5%) fibre coated with silver (Figure 1a,b). The second sample was a flocked yarn made by polyamide (PA) 6.6, with a liner density of 1.9 dTex (1.9 g per 10,000 m of fibre) and a diameter of 0.57 mm. In this case the core was made by 730 dTex PES (polyester) (96%) yarn and PA 6 (4%) fibre coated with carbon black (Figure 1a,c). No further information regarding the composition or production process was disclosed by the conductive yarn supplier, as these details are protected by industrial confidentiality. For clarity and readability throughout the manuscript, the silver-based flocked yarn will be referred to as F-Ag (flocked–silver), while the carbon black-based flocked yarn will be referred to as F-CB (flocked–carbon black).

Both samples were fabricated using electrostatic flocking technology. Aligned core yarns are tensioned, coated with a uniform film of water-based acrylic adhesive, and fed into a high-voltage flocking chamber. The flock springs up and down, moving along the lines of an electrostatic field generated by a high voltage system. The flock fibres are conductive and discharge themselves onto the water-based acrylic glue around the core. The wet flocked yarns, finally, pass through an oven where the glue is dried and recovered in a winding machine.

The weaving and finishing operations, necessary to obtain the fabric, were carried out by Apollo S.r.l. (Florence, Italy). The flocked yarn (2770 dTex between core structure and flock) is woven in weft together with a standard flat texturized polyester (FTF PES) yarn of 357 dTex count. The final weft density was approximately 140 weft/dm, constituted by two yarns of standard PES and one conductive flocked yarn. In warp a PES FTF 440 dTex count was used with a density of 320 yarns/dm. After weaving, the fabric was washed to remove residual oils and impurities, and thermally set using a stenter machine (rameuse) at 160 °C.

### 2.2. Methods

#### 2.2.1. Morphology and Structure by FESEM Analysis

Morphological characterisation of the samples was performed by FE-SEM TESCAN S9000G (Brno—Kohoutovice, Czech Republic) equipped with an Oxford Ultim-Max energy-dispersive X-ray spectroscopy (EDS) system, with AZtec software version AztecLive Advance 6.1 SP2, operating at 10 keV. SEM analysis was also employed to measure the mean diameter of the conductive fibres; a total of 20 measurements were performed on three different conductive fibres for each sample.

#### 2.2.2. TGA Analysis

Thermogravimetric analysis (TGA) was performed employing a TA Instrument Thermogravimetric Analyzer Discovery 550 (New Castle, DE, USA) from 30 °C up to 700 °C, under nitrogen (N_2_) flow, then to 800 °C in air and with a heating rate of 10 °C min^−1^. The gas flows applied in the balance and furnace sections were 40 mL min^−1^ and 60 mL min^−1^, respectively. About 12 mg of the sample was weighed in an alumina pan for analysis. This method was adopted to determine the polymer and carbon/Ag contents.

#### 2.2.3. DSC Analysis

Differential scanning calorimetry (DSC) analysis was carried out with a TA Instrument DSC Q200 equipment (New Castle, DE, USA) setting up a heat–cool–heat cycle to better understand the thermal properties of the conductive fibres. A total of 3 mg of the sample was placed in a hermetic aluminium pan and heated in an inert nitrogen atmosphere with flow of 50 mL/min from 10 to 290 °C at a heating and cooling rate of 10 °C/min. The degree of crystallinity (χ_*c*_) was calculated using the following Equation (1) [39]:

(1) $$\chi_{c}=\frac{{\Delta H}_{m}}{{\Delta H}_{m}^{0}\times \left(1-\emptyset \right)}\times 100$$

where *ΔH_m_* is the melting enthalpy of the sample; ${\Delta H}_{m}^{0}$ is the melting enthalpy of pure Nylon 6 with 100% crystallinity, equal to 230 J/g [40]; and ∅ is the weight fraction of fillers in the sample, measured by TGA. The melting enthalpy (*ΔH_m_*) was calculated by integrating the melting peak of the first heating ramp within the temperature ranges of 175 °C to 250 °C. In both TGA and DSC analyses, we focused exclusively on the conductive fibre, which consists only of polyamide and the conductive agent. The polyester core was not included in the analysed portion, as our aim was to characterise the thermal behaviour of the conductive layer specifically, being the functional component responsible for the electrical performance of the yarn.

#### 2.2.4. DC Electrical Measurements

To investigate the electrical properties, the conductive core of the flocked yarn was exposed by soaking the yarn extremity in acetone to attack the adhesive and remove the flock fibres.

Electrodes were prepared by applying RS PRO electrically conductive paint (Corby, UK) on the yarn ends and they were then were fixed on 3M™ Copper Foil Tape 1181 (St. Paul, MN, USA), to allow reliable electrical measurements and stable signal acquisition. Two-point DC resistance measurements were carried out using a Keithley integra series 2700 digital multimeter. The results were expressed as electrical resistance per unit length (RL), R = Ω/l, where Ω is the measured resistance and l (in cm) is the distance between the two electrodes. The measurements were performed at room temperature both on the samples before and after the different types of stresses and accelerated ageing described in Section 2.3.

### 2.3. Ageing Procedures

Samples were subjected to different types of mechanical and thermal ageing. Before the tests, all specimens were conditioned for 72 h in a standard atmosphere at 20 ± 2 °C with 65 ± 2% relative humidity, according to ISO 139.

#### Thermal Ageing

The first type of thermal stress was applied to reproduce medium heat and humidity conditions. It was simulated by the thermo-humid static chamber (CTUS), from the brand BAVA (Trofarello, Italy), subjecting the samples to 40 °C and 90% relative humidity, without condensation, for 250 h.

The second thermal stress simulated a condition of dry heat. The test was performed by keeping the samples at 90 °C for 48 h in a MEMMERT UF 450 (Schwabach, Germany) oven.

### 2.4. Mechanical Stress

The first type of mechanical stress was the determination of the wear resistance performed by a Cesconi abrasimeter brand Acquati (Arese, Italy). The purpose of this test was to simulate the rubbing of the clothes on the seat and the friction of the hands on the steering wheel. Samples were treated with six thousand revolution movements through standard abrading fabric with an applied load of 3 kg. The test was carried out on the samples dry, after soaking the fabrics in distilled water for 10 min and after CTUS (see Section Thermal Ageing). The resistance of the fibre to tensile tension was evaluated through a pulsating fatigue test performed on Acquati AG/7E/306 electronic dynamometer (Arese, Italy) according to ISO 13934-1. The test was executed starting with a preload of 200 N, running 1000 + 500 cycles both in warp/wale and weft/course directions. Different directions testing is necessary because fabric properties are affected by the loading direction [41].

The resistance of the fibres to bending after cold conditions was also tested. The sample was stationed at −30 °C for 6 h and conditioned at 23 °C folded on itself with a 2 kg load applied on it for 1 h.

### 2.5. Prototype Preparation

Concerning the preparation of the prototype, an Arduino MEGA 2560 microcontroller, manufactured by Arduino S.r.l. (Monza, Italy), was employed in combination with conventional electronic components, including resistors, light-emitting diodes (LEDs), a breadboard, jumper wires, and a USB cable.

## 3. Results

### 3.1. FE-SEM Analysis

**Figure 2:**
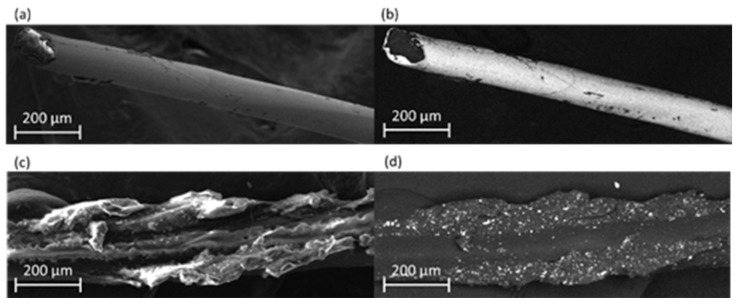
FE-SEM images of F-Ag and F-CB conductive fibres: (**a**) F-Ag conductive fibres using secondary electron (SE) detection; (**b**) F-Ag conductive fibres using back scattered electron (BSE) detection; (**c**) F-CB conductive fibres using secondary electron (SE) detection; and (**d**) F-CB conductive fibres using back scattered electron (BSE) detection.

The surface of F-Ag and F-CB conductive fibres was examined by FE-SEM to determine the fibre morphology and the distribution of the conductive coating on the polymer core. F-Ag exhibited a uniform conductive layer (Figure 2a), in particular, the backscattered electron (BSE) detection confirmed the presence of the metallic coating and provided additional evidence of its homogeneity across the fibre surface (Figure 2b) [42]. The uniformity of the coating is also demonstrated by the reproducible diameter of the F-Ag fibres, which was 50 µm, with a standard deviation of 3 µm. F-CB fibre showed, instead, an irregular distribution of the conductive coating (Figure 2c). BSE detection clearly revealed the internal structure of the PA fibre and the non-uniformity of the conductive layer (Figure 2d). In addition, EDS analysis detected the presence of aluminium as aluminium oxide (Al_2_O_3_), which can act as a dispersing agent to reduce the filler–filler interaction and enhance the filler–polymer interaction [43,44]. The results of EDS analysis are reported in Supplementary Materials in Figure S1. The presence of Al_2_O_3_ can explain the white spots visible in Figure 2d. As reported by Hwang et al. [45], CB concentrations exceeding 7 wt% can lead to surface irregularities and increased particle agglomeration. The CB aggregation and the presence of defects can contribute to decrease the electrical conductivity [46]. This also affects the measure of the fibre diameter which has a greater variability in the fibre size distribution. Indeed, the average diameter measured for the CB-based conductive fibres was 57.16 µm, with a standard deviation of 6 µm.

### 3.2. TGA Analysis

**Figure 3:**
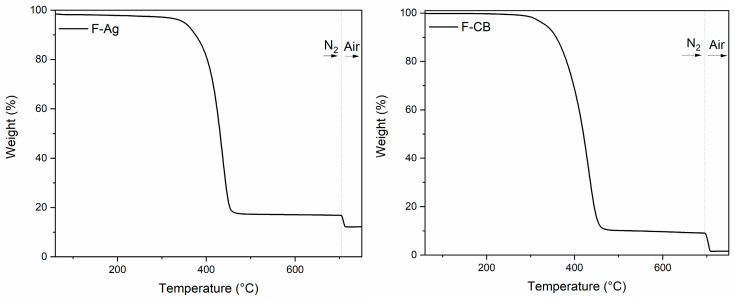
TGA curves of F-Ag and F-CB.

Thermogravimetric analysis (TGA) was performed to assess the thermal stability and estimate the relative composition of the two flocked fibres. In Figure 3, the TGA curves of F-Ag and F-CB can be observed.

F-Ag exhibited a single-stage decomposition process of the polymer fraction in nitrogen atmosphere with an onset degradation temperature of 403 °C and a maximum degradation rate at 436 °C. The 12% of residue found at 800 °C after opening in air is attributed to Ag. Also, F-CB showed the decomposition of the polymer in N_2_ atmosphere through a single step. The onset degradation temperature was set at 389 °C, with a maximum degradation rate at 430 °C. Carbon black remained stable above 500 °C; therefore, the residue observed provided an indication of the carbon black content, set at 9.4%. After opening the system to air at 800 °C, a residual 1.6% constituted by Al_2_O_3_ residue remained. In general, it resulted that the filler content, silver for F-Ag and carbon black for F-CB, increased the temperature of start degradation, which for bare PA 6 is around 377 °C [42]. In addition, the higher thermal stability of F-Ag is attributed to the presence of silver nanoparticles, which may exert a stabilising effect on the polymer matrix [42].

### 3.3. DSC Analysis

**Figure 4:**
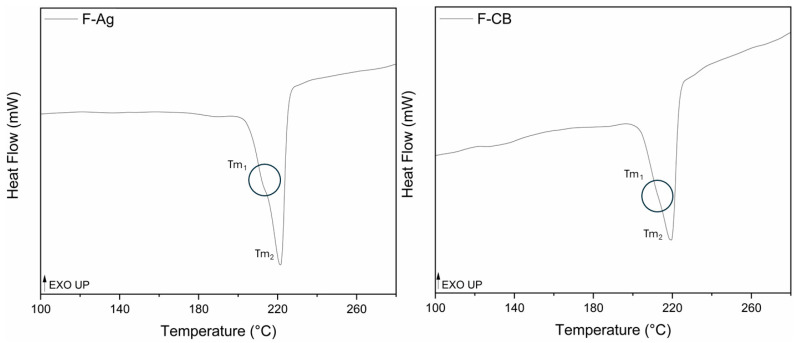
Second heating melting peaks of F-Ag and F-CB.

The results of DSC analysis are presented in Figure 4 that shows the second heating cycle of F-Ag and F-CB. The heat–cool–heat complete cycle of F-Ag and F-CB is reported in Figure S2 in Supplementary Materials. The broad and flat endothermic peak visible in the first heating, below 100 °C for both samples, indicated the presence of absorbed moisture and was eliminated during the second cycle. In F-Ag and F-CB, a melting peak at 223 °C, characteristic of the α-crystalline form of PA6, was observed, followed by a controlled cooling which results in one crystallization peak at 180 °C. During the second heating cycle a multi-phase melting peak (T_m_^1^ and T_m_^2^) was observed in both thermograms; T_m_^1^ is rounded by a circle in Figure 4. The main melting peak (T_m_^1^) at 220 °C for F-Ag and 218 °C for F-CB was attributed to the melting of the α-crystalline phase. The shoulder peak (T_m_^2^) at 212 °C, detected in F-Ag, and at 210 °C in F-CB, likely corresponded to the melting of the thermodynamically unstable γ-crystalline form [47,48]. In both samples the polymeric structure of the conductive yard is indeed confirmed to be polyamide 6.

The melting enthalpy and degree of crystallinity of both samples were reported in Table 1. The slightly higher crystallinity observed in F-Ag may be attributed to a more homogeneous coating and reduced interference with polymer chain mobility during crystallization. This is in line with the electrical conductivity results, which indicated better electrical performances for F-Ag. In contrast, F-CB presented a lower degree of crystallinity. This can be partially attributed to the inherent nature of carbon black, which, unlike metallic fillers, presents lower crystallinity. Additionally, the reduced crystallinity may result from the presence of particle aggregates that are unevenly dispersed throughout the polymer matrix. These aggregates hinder polymer chain mobility and orientation during crystallization, ultimately limiting crystal growth. The aggregates restricted the movement of the polymer chain and thereby limited crystal growth [39]. Moreover, the reduced crystallinity in F-CB aligned with the FESEM observations of surface irregularities and coating agglomeration, which negatively impacted the electrical behaviour. These findings highlight that not only the coating type but also its dispersion and interaction with the polymer matrix play a key role in determining the structural and functional performance of conductive fibres [49].

### 3.4. Electrical Measurements Before and After Stress

The electrical resistance per unit of length (RL) of F-Ag and F-CB was evaluated before and after different ageing processes and mechanical stresses. Considering the different types of conductive materials, different ranges of resistance values from a few ohms to many kiloohms, were collected (Table 2 and Table 3). Actually, the linear resistance of conductive tracks determined the resistance value of the electrical networks constructed within the fabric structure [16].

Before ageing, F-CB exhibited significantly higher RL [50], ranging from 31,944 to 24,222 Ω/cm, while F-Ag showed much lower RL values, between 5.9 and 3.2 Ω/cm. This difference is attributed to the intrinsic conductivity gap between carbon black and metallic silver, which is, respectively, 0.1 to 10^2^ S/cm [51] and 6.30 × 10^7^ S/m [52] at room temperature.

Both materials maintained good electrical stability after stress (Table 2 and Table 3), even if more pronounced variations in resistivity were registered for F-CB. Mechanical stresses such as dry wear, wet wear, and pulsed fatigue resulted in minor RL changes for both samples, generally within ±5% of the initial values.

In general, F-Ag demonstrated excellent stability under all tested conditions, showing robustness behaviour against thermal and mechanical degradation. In particular, under thermal cycling, no change in RL was observed for F-Ag, while F-CB showed slight fluctuations.

More aggressive stresses, such as thermal cycling and pulsed fatigue, resulted in slightly higher variations, particularly in F-CB, where RL increased marginally. In conclusion, F-Ag conductive fibre resulted to have lower RL and, consequently, a better electrical conductivity [53] before and after stress compared to R-CB.

### 3.5. Application: Multi-Touch Capacitive Sensor

**Figure 5:**
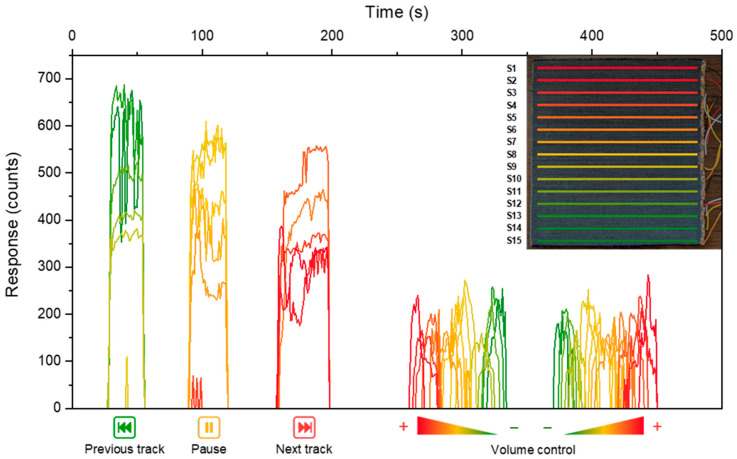
Sensors’ response to touch events over time. In the top-right corner a legend showing the disposition of the 15 sensors.

In this final section, we discuss the results obtained using this technology to produce a working prototype. Specifically, an Arduino MEGA 2560 microcontroller was employed to construct a music and media control interface, utilising the F-Ag as a capacitive multitouch sensor. F-Ag was selected for its better electrical properties. The setup comprises a 22 × 23 cm textile, in which silver-based conductive flocked yarns were grouped in sets of five and connected to the electronic circuitry using silver conductive paint, copper-based adhesive tape, and jumper wires. This configuration enabled the creation of 15 distinct capacitive sensors, equally spaced and electrically isolated from one another by approximately 7 mm (Figure 5). The principle underlying these sensors is based on measuring the time delay between the transmission of a digital signal from an Arduino’s digital pin and its reception on another, which is influenced by the capacitance present at the receiving pin. When this pin is connected to a large conductive surface, such as the flocked fabric, the user’s touch effectively introduces a second capacitive plate, increasing the time delay for the reception of the digital signal and allowing detection of the touch event [54]. The Arduino *Capacitive Sensing Library* [54] was employed to develop a control sketch, written in *JavaScript* and uploaded to the microcontroller via the *Arduino IDE*.

To ensure consistent and sensitive touch detection across all sensors, a calibration routine was implemented. Background values for each sensor were recorded over a 5-s interval using serial communication and averaged to establish baseline values. Subsequently, a 25 × 25 cm copper foil was placed over the textile to simulate simultaneous activation of all sensors. Activated values were again recorded over 5 s and averaged. The difference between activated and baseline values was then used to define an individual activation threshold for each sensor.

**Figure 6:**
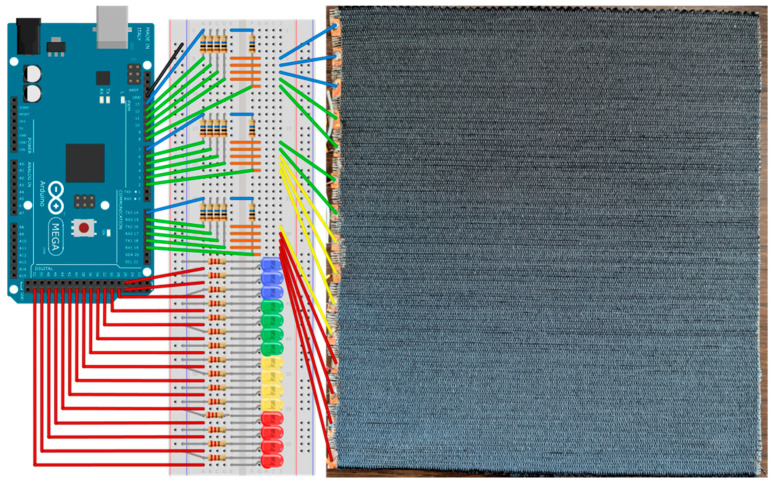
Experimental setup for capacitive multitouch sensing. The output is visually represented through a set of coloured LEDs (red, yellow, green, and blue) driven by the Arduino MEGA’s digital pins. This configuration allows real-time feedback of capacitive touch events.

A custom Python 3.10 script was developed to interface with the Arduino via serial communication, recording sensor data in CSV format, providing real-time visual feedback using the *matplotlib* library [55], and issuing media control commands to the host operating system via the *pyautogui* and *pycaw* libraries. As illustrated in Figure 6, different commands were assigned based on the region of fabric activated through touch input. When the lower five sensors were simultaneously triggered, the script issued a “previous track” command; the middle five sensors trigger a “play/pause” command, and the upper five sensors a “next track” command. Additionally, when individual sensors were touched sequentially, a volume control function was enabled, adjusting the system volume across 15 discrete levels (0–100%), each corresponding to a specific sensor.

The prototype also included 15 colour-coded LEDs to provide visual feedback, illuminating when the corresponding sensor exceeded its activation threshold. A demonstration video of the prototype in operation is available on Vimeo at the following link: https://vimeo.com/1089657691/b710e22583, accessed on 2 June 2025. Moreover, the possibility of employing carbon black-based (CB) flocked fabric as an alternative multitouch sensor was successfully investigated, with sensitivity and threshold values optimised accordingly.

## 4. Conclusions

In this study, two conductive flocked yarns incorporating different types of fillers—silver and carbon black—were comprehensively characterised in terms of morphology, thermal behaviour, and electrical stability under various ageing conditions. The silver-based yarn demonstrated a more uniform surface morphology, higher crystallinity, and superior conductivity, maintaining stable performance even after thermal and mechanical stress. In contrast, the carbon black-based sample exhibited greater variability in resistivity and reduced crystallinity, likely due to filler agglomeration and inhomogeneous dispersion.

Despite the performance gap, carbon black remains a viable, low-cost alternative with potential for improvement through optimisation of filler dispersion and surface engineering. Furthermore, the successful implementation of the silver-based fabric in a functional multitouch sensor highlights the feasibility of integrating these materials into flexible, interactive systems for automotive interiors.

These findings support the adoption of conductive flocked fabrics as a promising platform for low-voltage, durable e-textile applications. Future research will focus on enhancing the durability and performance of carbon-based formulations and exploring scalable fabrication processes compatible with automotive manufacturing standards. Future research will focus on enhancing the durability and performance of carbon-based formulations and explore scalable fabrication processes compatible with automotive manufacturing standards. Additionally, the development of graphite-based conductive flocked yarns represents a promising avenue, as graphite offers excellent electrical conductivity and could serve as a balanced compromise between the high performance of silver coatings and the cost-effectiveness of carbon black.

Regarding the silver-based prototype, an interesting dual-function application could be the exploitation of its well-known antibacterial properties. It may be possible to combine conductivity and antibacterial functionality in components such as armrests, enabling hidden interactive features (e.g., control of interior lighting, audio volume, or media playback) while contributing to increasing hygiene and safety in the vehicle interior. Furthermore, such materials could be integrated into air filtration systems, where variations in textile conductivity might serve as indicators of pathogen concentration, offering a novel approach to monitoring filter efficiency in automotive HVAC units.

## Figures and Tables

**Table 1:** Thermal properties of F-Ag and F-CB conductive fibres obtained from DSC analysis.

Sample	T_m_ [°C] First Heating	T_m_^1^ [°C] Second Heating	T_m_^2^ [°C] Second Heating	Melting Enthalpy (*ΔH_m_*) Measured [J/g]	Degree of Crystallinity (χ_*c*_)
F-Ag	223	220	212	57.2	31
F-CB	223	228	210	52.1	25

**Table 2:** Electrical measurement results of F-Ag before and after the various thermal and mechanical ageing procedures. For each specimen, the length between the electrodes (l) and the corresponding RL value are reported, followed by the same measurements after heat ageing, combined thermos-humid ageing (CTUS), thermal cycling, dry/wet abrasion, CTUS + wear, cold bending, and pulsed fatigue in longitudinal (L) and transverse (T) directions.

F-Ag						
Before Ageing/Stress			After Ageing/Stress			
Sample	l [cm]	RL [Ω/cm]	Sample	Type of Ageing/Stress	l [cm]	RL [Ω/cm]
1	24.8	3.2	1	Heat ageing	24.8	3.1
2	24	4.5	2	CTUS	24	4.5
3	6	5.6	3	Thermal cycles	6	5.6
4	18.7	4.5	4	Dry wear	18.7	4.6
5	19	5.0	5	Wet wear	19	4.9
6	19.5	4.6	6	CTUS+wear	19.5	4.5
7	12.5	4.9	7	Cold bending	12.5	4.9
8	27	5.9	8	Pulsed fatigue L	27	5.9
9	11.5	4.6	9	Pulsed fatigue T	11.5	4.5

**Table 3:** Electrical measurement results of R-CB before and after the various thermal and mechanical ageing procedures. For each specimen, the length between the electrodes (l) and the corresponding RL value are reported, followed by the same measurements after heat ageing, combined thermo-humid ageing (CTUS), thermal cycling, dry/wet abrasion, CTUS+wear, cold bending, and pulsed fatigue in longitudinal (L) and transverse (T) directions.

F-CB						
Before Ageing/Stress			After Ageing/Stress			
Sample	l [cm]	RL [Ω/cm]	Sample	Type of Ageing/Stress	l [cm]	RL [Ω/cm]
1	28	25,482	1	Heat ageing	28	27,679
2	27	24,222	2	CTUS	27	24,907
3	6	18,333	3	Thermal cycles	6	18,333
4	19	26,526	4	Dry wear	19	26,842
5	20	27,350	5	Wet wear	20	27,850
6	18	31,944	6	CTUS+wear	18	30,500
7	12	28,417	7	Cold bending	12	28,167
8	27	26,704	8	Pulsed fatigue L	27	26,852
9	11	28,818	9	Pulsed fatigue T	11	28,909

## Data Availability

The original contributions presented in the study are included in the article and Supplementary Materials; further inquiries can be directed to the corresponding authors.

## Supplementary Materials

The supporting information can be downloaded as follows: https://www.mdpi.com/article/10.3390/polym17162212/s1, Figure S1: EDS analysis of sample F-CB; Figure S2: Heat-Cool-Heat DSC curves of F-Ag and F-CB.
