# Supplementary material for: Investigation of Composition, Structure, Electrical Properties, and Ageing Resistance of Conductive Flocked Fabric for Automotive Applications
Source: Polymers (Basel). 2025 Aug 13;17(16):2212. doi: 10.3390/polym17162212 (PMC12390296; doi:10.3390/polym17162212)
Supplement: Supplementary file 1 [file polymers-17-02212-s001.zip › polymers-3773550-supplementary.pdf]

polymers

MDPI

Supplementary Materials

Investigation of Composition, Structure, Electrical Properties, and Ageing Resistance of Conductive Flocked Fabric for Automotive Applications

Matilde Arese [1,†], Elio Sarotto [1,†], Antonino Domenico Veca [2], Vito Guido Lambertini [2], Daniele Nardi [3], Martina Sandigliano [4], Federico Cesano [1] and Valentina Brunella [1,*]

[1] Department of Chemistry, University of Turin, 10125 Turin, Italy; matilde.arese@unito.it (M.A.); elio.sarotto@unito.it (E.S.); federico.cesano@unito.it (F.C.)
[2] Fiat Research Center SCPA (CRF), Stellantis, 10135 Turin, Italy; antonino.veca@crf.it (A.D.V.); vitoguido.lambertini@crf.it (V.G.L.)
[3] Apollo Srl, 50013 Campi Bisenzio, Italy; daniele.nardi@sageai.com
[4] Technova Srl, 07026 Olbia, Italy; martina.sandigliano@technovaitalia.it
* Correspondence: valentina.brunella@unito.it; Tel.:+39-011-6707546
† These authors contributed equally to this work.

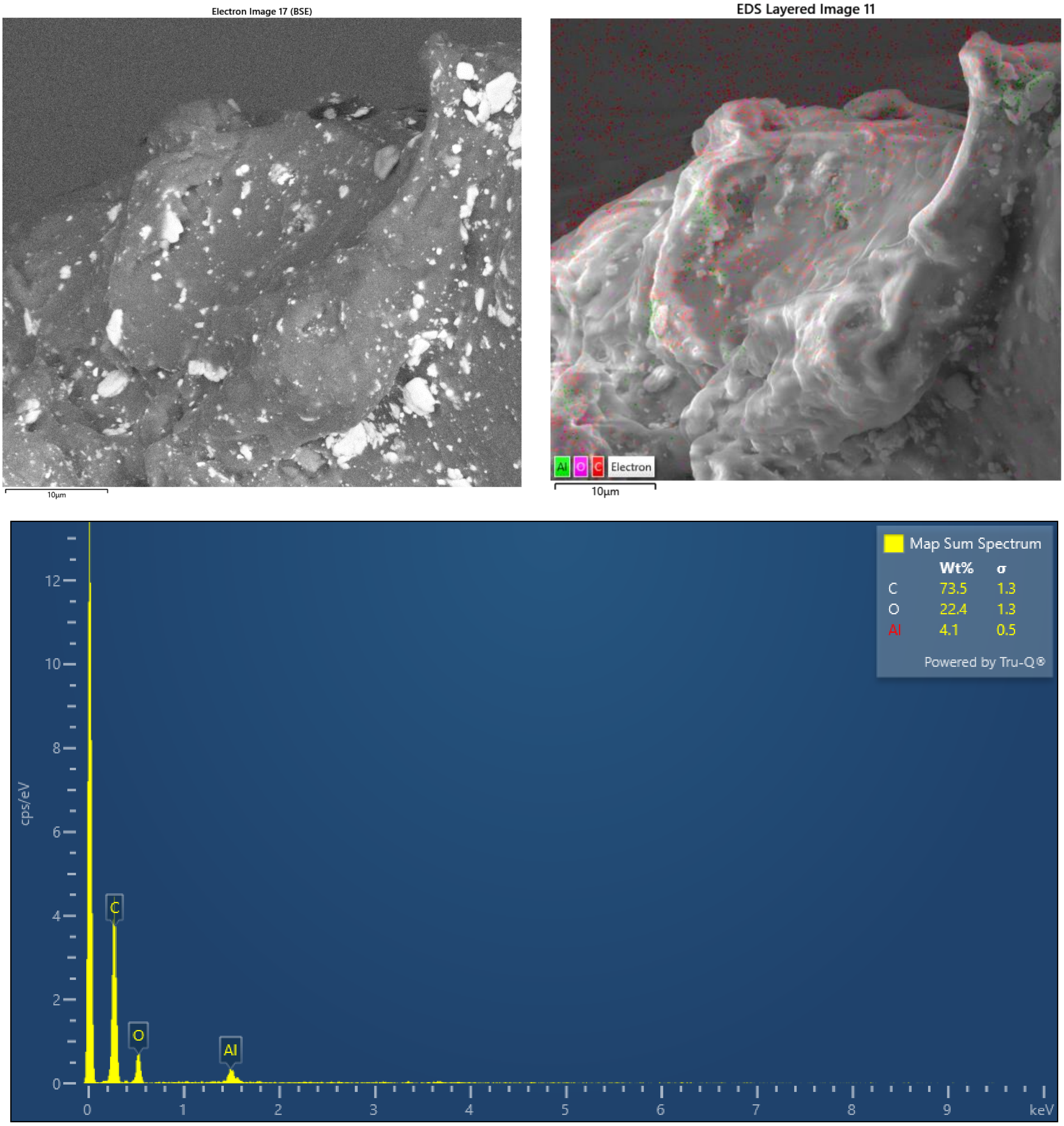

Figure. S1 EDS analysis of sample F-CB

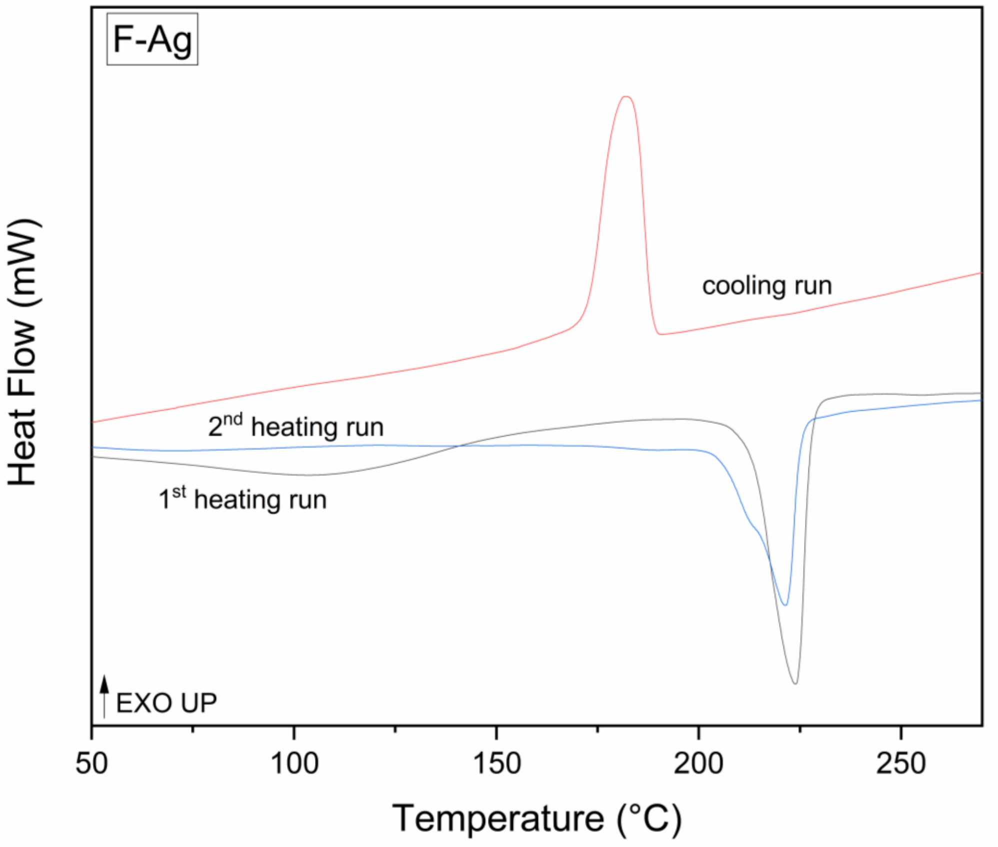

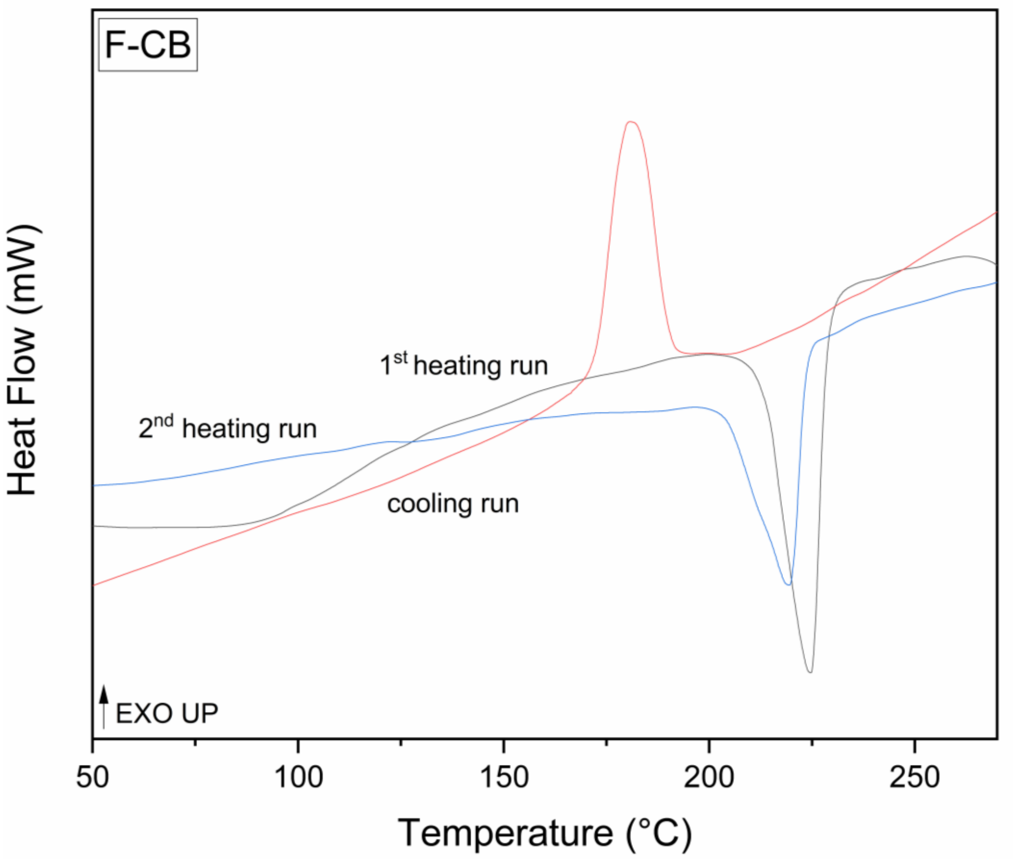

Figure. S2 Heat-cool-heat DSC curves of F-Ag and F-CB. 1st heating run is in black colour, cooling run is in red colour, 2nd heating run is in blue colour.
